# Beyond Binary ICU Admission: Duration-Sensitive Evaluation and Patient-Level Reclassification of Textbook Outcome After Colorectal Cancer Surgery

**DOI:** 10.3390/jcm15176809

**Published:** 2026-09-02

**Authors:** Adem Ozcan, Gizem Gunes, Ali Bal, Abdulkadir Unsal

**Affiliations:** Department of Surgical Oncology, Ankara Bilkent City Hospital, Ankara 06800, Türkiye

**Keywords:** colorectal cancer, textbook outcome, intensive care unit, composite quality measure, patient-level reclassification, surgical benchmarking, postoperative outcomes

## Abstract

**Background/Objectives:** Textbook outcome (TO) is an all-or-none composite in which a common, practice-sensitive component may exert disproportionate influence on patient classification. We evaluated the classification impact of binary postoperative intensive care unit (ICU) admission and the additional information retained by actual ICU duration after colorectal cancer surgery. **Methods:** This retrospective single-center cohort included 241 patients undergoing colorectal cancer resection between 2020 and 2026. Comprehensive TO required no major postoperative complication, reoperation, 30-day mortality, or postoperative ICU admission, together with microscopically margin-negative (R0) resection of the colorectal primary and postoperative length of stay ≤14 days. Core TO omitted ICU admission and length of stay and was used as an analytical comparator rather than as a newly validated TO definition. Patient-level reclassification was the primary outcome. ICU duration was evaluated continuously in the principal concurrent-association analysis; the 0-, 1-, 2-, and ≥3-day categories were exploratory. **Results:** Comprehensive and core TO were achieved in 27 (11.2%) and 169 (70.1%) patients, respectively, resulting in reclassification of 142 patients (58.9%; exact McNemar *p* < 0.001). ICU admission alone accounted for reclassification in 118 patients (49.0% of the cohort). Each additional ICU day showed a concurrent association with higher odds of core TO failure (adjusted odds ratio (OR), 1.43; 95% confidence interval (CI), 1.25–1.63) and major postoperative complications (adjusted OR, 1.63; 95% CI, 1.40–1.91; both *p* < 0.001). Categorical estimates for 1- and 2-day stays were imprecise and did not establish equivalence with no ICU admission. Duration-sensitive representations showed better internal model fit, discrimination, and probabilistic performance than binary ICU admission. **Conclusions:** In this center with frequent postoperative ICU utilization, binary ICU admission dominated comprehensive TO classification. ICU duration retained additional clinical information, but the findings do not establish the intrinsic invalidity of the ICU component, equivalence of short stays with no ICU admission, or a universal duration cutoff. Multicenter external validation across different ICU policies is required.

## 1. Introduction

Textbook outcome (TO) offers an intuitively simple answer to a difficult question: did a patient experience an ideal episode of colorectal cancer surgery? Introduced as an all-or-none composite, TO condenses oncological adequacy, perioperative safety, and postoperative recovery into a single patient-level endpoint [1]. Its clinical relevance is supported by prospective and trial-derived evidence associating TO achievement with improved long-term outcomes after colorectal cancer resection [2,3]. Yet the same architecture that makes TO concise also creates its central methodological vulnerability: every component has identical veto power over the final classification, even when components differ substantially in clinical severity, importance to patients, and susceptibility to local care pathways [4,5].

For a metric intended to support quality assessment and institutional benchmarking, this vulnerability is consequential. A systematic review and meta-analysis of 15 colorectal studies comprising 301,502 patients reported a pooled TO achievement rate of 55%, while identifying no standardized definition [6]. Subsequent colorectal-specific and pan-oncological systematic reviews confirmed substantial variation in complication thresholds, length-of-stay criteria, readmission, stoma-related outcomes, oncological adequacy, and intensive care unit (ICU) utilization [7,8]. Even expert consensus has yielded a broad, context-sensitive construct rather than a single, readily transportable standard [9]. Consequently, variation in reported TO rates may reflect not only differences in surgical performance, but also the selection and operationalization of individual components.

Postoperative ICU admission provides a particularly revealing test of this problem. Some contemporary colorectal TO definitions classify any postoperative ICU admission as failure, whereas others omit ICU utilization altogether [2,3,6,7,8,9]. However, ICU admission after colorectal cancer surgery is not a homogeneous clinical event. It may be planned for enhanced surveillance after major surgery or occur unexpectedly in response to postoperative deterioration, and the clinical factors associated with planned and unplanned ICU transfer differ [10]. More broadly, postoperative ICU utilization varies considerably across hospitals and does not demonstrate a uniform relationship with postoperative outcomes after case-mix adjustment [11,12]. Recent population-based colon cancer data further show that unplanned ICU admission—particularly when associated with surgical reintervention and a longer ICU stay—identifies a distinctly high-risk postoperative course [13]. A binary component that assigns the same failure status to brief ICU utilization and prolonged critical-care treatment may therefore lack the specificity required for an unqualified all-or-none quality criterion.

To our knowledge, no previous colorectal TO study has quantified patient-level reclassification attributable to binary ICU admission while directly evaluating the additional information retained by actual postoperative ICU duration. The present study therefore examined how inclusion of any postoperative ICU admission affected patient-level TO classification and whether actual ICU duration provided additional concurrent clinical information. We compared two nested, protocol-defined constructs: a comprehensive TO incorporating ICU admission and prolonged hospitalization, and a core TO restricted to major postoperative complications, reoperation, 30-day mortality, and microscopically margin-negative resection of the colorectal primary. We hypothesized that, in a setting of frequent postoperative ICU utilization, binary ICU admission would exert substantial influence on TO classification and that actual ICU duration would retain more clinical information than a yes/no representation.

## 2. Materials and Methods

### 2.1. Study Design and Setting

This retrospective, single-center observational cohort study was conducted at the Surgical Oncology Clinic of Ankara Bilkent City Hospital, Ankara, Türkiye. All eligible adult patients who underwent resection of a colorectal primary between 1 January 2020 and 1 May 2026 were assessed. The surgical accrual window closed before ethics approval. The study protocol was approved on 6 May 2026, before any study-specific data abstraction, analytical database construction, or statistical analysis was initiated. Accordingly, the cohort comprised historical routinely generated clinical records; no post-approval or simulated patient record was included. The study was reported in accordance with the Strengthening the Reporting of Observational Studies in Epidemiology (STROBE) statement [14]. Because the study used a fixed all-eligible cohort, no a priori sample-size calculation was performed.

### 2.2. Ethical Approval and Data Governance

The study was conducted in accordance with the Declaration of Helsinki and approved by the Ankara Bilkent City Hospital Medical Research Scientific and Ethical Evaluation Board (approval No. TABED 1-26-2537; 6 May 2026).

Direct identifiers were removed before analysis, and each patient was assigned a unique study code. The re-identification key was stored separately within the institution and was not included in the analytical file. Access to identifiable source records and the re-identification key was restricted to the ethics-approved investigators. Study variables were abstracted from institutional source records, and any discrepancy was resolved by direct review of the electronic medical record, operative report, postoperative ICU record, discharge documentation, or finalized pathology report before database lock. Data abstraction and analysis were initiated after ethics approval.

### 2.3. Patient Selection

Patients were eligible for inclusion when they met all of the following criteria:

1. age ≥ 18 years at the time of surgery;
2. histopathologically confirmed colorectal adenocarcinoma;
3. surgical resection of the colorectal primary during the approved study period;
4. availability of the perioperative information required to determine the protocol-defined textbook outcome components.

Both curative- and palliative-intent primary-tumor resections were eligible, and synchronous distant metastatic disease was not an exclusion criterion, in accordance with the approved real-world cohort protocol. These patients were retained to preserve the protocol-defined real-world cohort. To evaluate the influence of metastatic heterogeneity, the principal continuous-duration analyses were repeated after restriction to patients without synchronous metastatic disease.

Patients were excluded for benign final pathology, histology other than colorectal adenocarcinoma, a synchronous second primary malignancy, or missing data that prevented determination of either primary TO construct. Duplicate institutional records were reconciled through source review, and each patient was represented once using the index colorectal resection.

### 2.4. Data Sources and Data Quality

Data were retrospectively obtained from the institutional hospital information management system, inpatient and outpatient records, operative reports, anesthesia documentation, postoperative ICU records, discharge summaries, and finalized pathology reports.

A predefined data dictionary was used during data extraction. Before analysis, the database underwent patient-level logical checks involving:

- consistency between 30-day mortality and the highest recorded Clavien–Dindo grade;
- postoperative length of stay;
- reoperation and Clavien–Dindo severity;
- postoperative ICU admission and cumulative ICU duration;
- resection-margin status;
- harvested and metastatic lymph-node counts.

When an inconsistency was identified, the original source record was reviewed and the verified value was retained. The analytical database was locked after completion of all predefined checks.

### 2.5. Clinical, Operative, and Pathological Variables

Recorded baseline variables included age, sex, American Society of Anesthesiologists physical status (ASA), Eastern Cooperative Oncology Group performance status (ECOG), and the presence of diabetes mellitus, hypertension, coronary artery disease, and chronic obstructive pulmonary disease.

Tumor-related variables included primary tumor location, pretreatment clinical T category, postoperative nodal category, synchronous metastatic status, lymphovascular invasion, perineural invasion, the number of examined lymph nodes, and the number of metastatic lymph nodes. Clinical T category was recorded from the pretreatment staging assessment. Postoperative nodal status was recorded as pN in patients undergoing upfront surgery and as ypN in patients undergoing resection after neoadjuvant treatment. Staging terminology followed the eighth edition of the American Joint Committee on Cancer tumor–node–metastasis classification [15].

The tumor-dimension variable represented the maximum dimension reported in the finalized pathology report. In patients with ypT0 pathological complete response, any non-zero dimension recorded in the pathology report was regarded as the reported tumor-bed or fibrotic-scar dimension and was not described or analyzed as residual viable tumor size.

Operative variables included emergency versus elective operative status, surgical approach, type of colorectal resection, and stoma formation during the index operation. Calendar year of surgery was recovered from the source-verified chronological cohort record and retained as a 2020–2026 variable; exact operation dates were not included in the analytical dataset. Surgical approach was categorized as open or laparoscopic. Stoma formation was treated as an operative characteristic rather than an automatic textbook outcome failure because it could represent either an integral component of the index operation or an intended protective strategy.

Resection status referred specifically to the colorectal primary specimen. R0 primary-tumor resection was defined as microscopically margin-negative resection of the colorectal primary, irrespective of the presence of synchronous distant metastatic disease. R1 indicated microscopic involvement of a resection margin. No assumption regarding clearance of distant metastatic disease was derived from the primary-specimen margin status.

Circumferential resection-margin distance was recorded for rectal tumors when explicitly reported in the finalized pathology report. Missing circumferential-margin values were retained as true missing data; no value was inferred from the overall R0 status and no imputation was performed.

### 2.6. Postoperative Outcomes

Postoperative complications occurring during the index hospitalization or within 30 days after surgery were classified according to the Clavien–Dindo system [16]. The highest documented grade experienced by each patient during the 30-day postoperative period was used.

Grade I represented a documented deviation from the expected postoperative course that did not require surgical, endoscopic, or radiological intervention or pharmacological treatment beyond the therapies permitted within Grade I. Routine prophylactic antiemetic administration and standard postoperative analgesia alone were not considered complications. A major postoperative complication was defined as Clavien–Dindo grade III or higher. Because reoperation and grade V mortality could also satisfy the Clavien–Dindo grade ≥ III criterion, structural overlap among core TO components was expected. The primary analysis preserved the approved Clavien–Dindo grade ≥ III definition, while a sensitivity analysis restricted nonfatal major postoperative complications to grades III–IV and retained 30-day mortality as a separate component.

Reoperation was defined as an unplanned return to the operating room for a postoperative complication within 30 days of the index operation. Thirty-day mortality was defined as death from any cause within 30 days after surgery, regardless of whether death occurred during the index hospitalization or following discharge.

Postoperative length of stay (LOS) was calculated from the date of the index operation to hospital discharge or in-hospital death. Prolonged hospitalization was defined in the primary analysis as a postoperative length of stay exceeding 14 days.

### 2.7. Postoperative ICU Utilization

Postoperative ICU admission was recorded when a patient was admitted or transferred to an ICU following the index operation. ICU duration was defined as the cumulative number of calendar days spent in postoperative intensive care after the index procedure. Preoperative ICU exposure was not included. Patients without postoperative ICU admission were assigned a duration of 0 days, while same-day or overnight postoperative ICU observation was recorded as 1 day.

ICU admission was retained as a binary variable for the protocol-defined comprehensive textbook outcome. Actual ICU duration was evaluated separately to determine whether brief and prolonged ICU utilization showed different concurrent associations with clinically consequential postoperative outcomes.

For descriptive presentation, postoperative ICU duration was grouped as 0, 1, 2, and ≥3 calendar days. The ≥3-day category pooled sparse longer-duration observations and was treated as exploratory; it was not selected through outcome optimization and was not interpreted as a validated or universal cutoff. ICU duration was analyzed principally as a continuous variable and secondarily using a natural cubic spline.

Planned or prophylactic postoperative ICU monitoring could not be distinguished consistently from unplanned ICU admission or transfer prompted by postoperative deterioration. ICU indication was therefore unavailable as a patient-level variable, and duration was analyzed only as a quantitative representation of ICU utilization, not as a surrogate for admission intent, physiological severity, or the appropriateness of ICU care.

### 2.8. Textbook Outcome Definitions

Two nested, protocol-defined TO constructs were evaluated. For clarity, the protocol-defined classical and modified definitions are referred to throughout the manuscript as comprehensive TO and core TO, respectively. Core TO was used solely as a minimal analytical comparator to isolate the classification impact of ICU admission and prolonged hospitalization. It was not intended as a complete measure of oncological quality or as a validated replacement for existing TO definitions. Its R0 component referred exclusively to microscopically negative margins of the colorectal primary specimen and did not imply clearance of synchronous metastatic disease.

#### 2.8.1. Core Textbook Outcome

Core textbook outcome required the simultaneous fulfillment of all of the following criteria:

1. no major postoperative complication, defined as Clavien–Dindo grade < III;
2. no reoperation within 30 days;
3. no death within 30 days;
4. R0 resection of the colorectal primary.

Failure of any individual component resulted in core TO failure.

#### 2.8.2. Comprehensive Textbook Outcome

Comprehensive textbook outcome required fulfillment of all core TO components together with:

5. no postoperative ICU admission;
6. no prolonged hospital stay, defined as postoperative length of stay ≤ 14 days.

Both constructs were analyzed using an all-or-none approach. No component was weighted, and failure of any included component resulted in failure of the corresponding composite.

### 2.9. Patient-Level Reclassification

The nested definitions permitted assignment of each patient to one of three mutually exclusive classification categories:

#### 2.9.1. Concordant TO Achievement

Patients who fulfilled both the comprehensive TO and core TO definitions.

#### 2.9.2. Care-Pathway-Related Reclassification

Patients who fulfilled all core TO criteria but failed comprehensive TO exclusively because of postoperative ICU admission, prolonged hospitalization, or both. This category was further divided descriptively into:

- ICU-only reclassification;
- length-of-stay-only reclassification;
- combined ICU-plus-length-of-stay reclassification.

#### 2.9.3. Core TO Failure

Patients who failed at least one core TO component because of major postoperative complication, reoperation, 30-day mortality, or non-R0 resection of the colorectal primary.

The reclassification analysis described how inclusion of the two care-pathway components changed patient classification; it did not redefine a clinically adverse core outcome as a successful postoperative course.

### 2.10. Component-Sensitivity Analyses

The influence of each comprehensive TO component was evaluated using component-attainment, sole-component contribution, and leave-one-component-out analyses. A sole-component contribution was present when a patient failed exactly one component while fulfilling every other comprehensive TO criterion. This analysis quantified classification behavior under the observed component prevalence and was not interpreted as a direct measure of clinical importance or construct validity.

To examine the sensitivity of TO achievement to alternative operational representations of ICU utilization, the following scenarios were reported jointly:

1. strict comprehensive TO requiring fulfillment of all core TO criteria, 0 ICU days, and postoperative LOS ≤ 14 days;
2. a duration-sensitive scenario requiring fulfillment of all core TO criteria, ICU duration ≤ 1 day, and postoperative LOS ≤ 14 days;
3. a duration-sensitive scenario requiring fulfillment of all core TO criteria, ICU duration ≤ 2 days, and postoperative LOS ≤ 14 days;
4. a hybrid scenario requiring fulfillment of all core TO criteria and postoperative LOS ≤ 14 days, with the ICU component omitted;
5. core TO, with both ICU utilization and LOS omitted.

These scenarios were used exclusively as definition-sensitivity analyses. No alternative scenario was selected or promoted on the basis of the resulting achievement rate, and none was considered a validated replacement for existing TO constructs.

### 2.11. Study Outcomes

The primary outcome was patient-level reclassification attributable to inclusion of postoperative ICU admission and prolonged hospitalization in the comprehensive TO construct.

Key secondary analyses evaluated:

1. the paired difference between comprehensive and core TO achievement rates;
2. component-specific attainment and sole-component contributions to reclassification;
3. the concurrent association of continuous postoperative ICU duration with core TO failure and major postoperative complications;
4. the internal fit, discrimination, and probabilistic performance of binary and duration-sensitive ICU representations.

Exploratory analyses evaluated outcomes across categorical ICU-duration groups; the ≥3-day versus 0–2-day representation; alternative LOS thresholds; Firth penalized estimates; a nonfatal Clavien–Dindo grade III–IV endpoint; reclassification within M0 disease (absence of synchronous distant metastatic disease), colon cancer, rectal cancer, open surgery, and laparoscopic surgery subgroups; and expansion of the adjustment set using ASA class, emergency operative status, calendar year, and surgical approach.

ICU duration and postoperative complications arose during the same postoperative episode. The timing of complication onset relative to ICU admission, transfer, or discharge could not be determined consistently from the retrospective records. ICU duration was therefore treated as a concurrent marker of the postoperative course rather than as an antecedent exposure. The regression coefficients were intended to evaluate clinical interpretability and information retention and cannot support etiological, causal, or preoperative predictive inference.

### 2.12. Statistical Analysis

Continuous variables were assessed using distributional plots and the Shapiro–Wilk test and were summarized as mean ± standard deviation or median and interquartile range, as appropriate. Categorical variables were reported as numbers and percentages. Variable-specific denominators were used when data were not applicable or were genuinely missing.

Component-attainment and TO-achievement proportions were reported with Wilson 95% confidence intervals. Because comprehensive and core TO were evaluated in the same patients and the constructs were nested, their paired achievement rates were compared using the exact McNemar test. The patient-level reclassification proportion and sole-component contribution frequencies were reported descriptively with corresponding confidence intervals.

Exploratory comparisons across concordant TO achievement, care-pathway-related reclassification, and core TO failure used the Kruskal–Wallis test for continuous variables and the Pearson chi-square or an exact contingency-table test for categorical variables, as appropriate. These classification-category comparisons were exploratory and supportive; no post hoc pairwise comparisons were performed, and no multiplicity adjustment was applied.

Outcomes across the descriptive ICU-duration categories of 0, 1, 2, and ≥3 days were compared using the Kruskal–Wallis test for continuous variables, the Pearson chi-square test for adequately populated categorical tables, and the Fisher–Freeman–Halton exact test for sparse tables.

Two otherwise identical case-mix-adjusted logistic regression models were fitted separately for core TO failure and major postoperative complications. The binary model represented ICU utilization as any versus no postoperative ICU admission. The duration-sensitive model used indicator variables for 1 day, 2 days, and ≥3 days, with 0 days as the reference category. The original adjustment set included age per 10-year increase, ECOG performance status ≥ 2, synchronous metastatic disease, tumor perforation, and tumor-related obstruction.

The original adjustment set was deliberately parsimonious because the two outcomes comprised 72 and 54 events, respectively. It represented age, functional status, metastatic burden, and acute tumor-related presentation and was selected before model fitting without automated stepwise procedures. Exploratory sensitivity models added ASA class ≥ III, emergency operative status, calendar year, and surgical approach individually or in clinically relevant combinations. Calendar year was modeled continuously. The continuous-duration analyses were also repeated after restriction to patients with M0 disease.

A conceptual directed acyclic graph illustrating the assumed relationships among baseline patient factors, acute tumor-related presentation, operative characteristics, center-level ICU policy, evolving postoperative severity, ICU utilization, and postoperative outcomes is provided in Supplementary Figure S4. The graph treats postoperative severity as a common determinant of ICU duration and adverse outcomes and does not assume a causal pathway from ICU duration to postoperative complications. Multicollinearity was assessed using variance inflation factors.

Binary and duration-sensitive models were compared using the likelihood-ratio test, Akaike information criterion (AIC), area under the receiver-operating-characteristic curve (AUC), and Brier score. These measures were used to compare the information retained by the ICU representations and not to develop a clinical prediction tool. Optimism in AUC estimates was assessed using 500 bootstrap attempts; the number of successfully fitted bootstrap samples was reported. For each successfully fitted bootstrap sample, optimism was calculated as the difference between the AUC within the bootstrap sample and the AUC obtained when the bootstrap-fitted model was applied to the original cohort; mean optimism was then subtracted from the apparent AUC. Adjusted marginal probabilities were standardized over the observed covariate distribution, and 95% confidence intervals were obtained by parametric simulation using 5000 coefficient draws from the fitted model.

Sensitivity analyses modeled ICU duration continuously per additional day and using a natural cubic spline with three degrees of freedom. Overall association and departure from linearity were evaluated using likelihood-ratio testing. Because sparse outcome cells produced wide maximum-likelihood estimates, bias-reduced Firth penalized logistic regression was used as a sensitivity analysis [17]. A further sensitivity analysis defined nonfatal major postoperative complications as Clavien–Dindo grades III–IV, leaving 30-day mortality as a separate component. The ICU ≥3-day versus 0–2-day analysis was exploratory and was not interpreted as establishing a universal cutoff. Additional exploratory analyses evaluated LOS thresholds of 10, 14, and 15 days and reclassification within M0 disease, colon cancer, rectal cancer, open surgery, and laparoscopic surgery subgroups.

Primary TO variables were complete. Missing secondary pathological variables, including circumferential resection-margin distance, were analyzed using available cases without imputation. The paired reclassification analysis was the primary analysis. Continuous ICU duration and comparison of binary versus duration-sensitive representations were designated as key secondary analyses. ICU-duration categories, the ≥3-day grouping, alternative LOS thresholds, Firth models, subgroup analyses, expanded covariate sets, and calendar-time analyses were exploratory. No formal multiplicity correction was applied to these secondary and exploratory analyses; their estimates were interpreted according to effect size, confidence-interval width, internal consistency, and clinical plausibility rather than statistical significance alone. All tests were two-sided, and *p* < 0.05 was considered statistically significant. Statistical analyses were performed using IBM SPSS Statistics for Windows, version 30.0 (IBM Corp., Armonk, NY, USA), and Python version 3.13.5. Python-based analyses were conducted using statsmodels version 0.14.6, SciPy version 1.17.0, and scikit-learn version 1.8.0.

## 3. Results

### 3.1. Study Cohort

**Figure 1:**
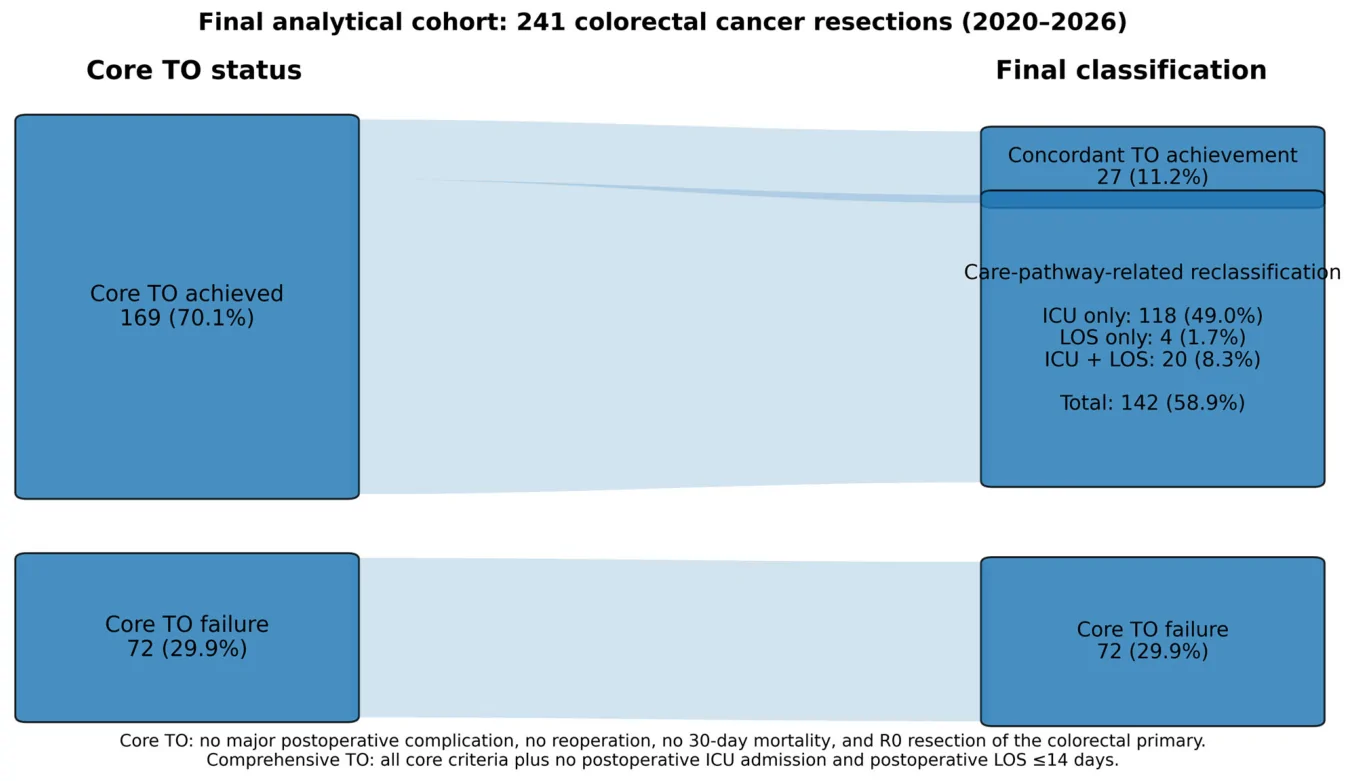
Patient-level reclassification across the nested textbook outcome constructs. Flow widths are proportional to the number of patients in each pathway. Of 241 patients, 169 achieved core TO. Among these patients, 27 also achieved comprehensive TO and were classified as having concordant TO achievement, whereas 142 were reclassified by the care-pathway components. The reclassified group comprised 118 ICU-only, 4 LOS-only, and 20 combined ICU-plus-LOS reclassifications. The remaining 72 patients had core TO failure. TO, textbook outcome; ICU, intensive care unit; LOS, length of stay.

The final analytical cohort comprised 241 patients who underwent colorectal cancer resection between 2020 and 2026 at the Surgical Oncology Clinic of Ankara Bilkent City Hospital. All variables required to determine the protocol-defined textbook outcome constructs and postoperative intensive care unit (ICU) duration were complete. The final analytical cohort and patient-level classification across the nested textbook outcome definitions are summarized in Figure 1, while the demographic, clinical, operative, pathological, and postoperative characteristics of the cohort are presented in Table 1.

The median age was 66 years (interquartile range [IQR], 55–72), and 165 patients (68.5%) were male. An Eastern Cooperative Oncology Group performance status ≥ 2 was present in 83 patients (34.4%), while 45 patients (18.7%) had an American Society of Anesthesiologists class ≥ III. Tumor-related obstruction and perforation were documented in 68 (28.2%) and 41 (17.0%) patients, respectively.

Rectal tumors accounted for 106 cases (44.0%). Clinical T3–4 disease was present in 198 patients (82.2%), final pathological nodal involvement in 108 (44.8%), and synchronous metastatic disease in 27 (11.2%). The median maximum dimension reported in the final pathology report was 43 mm (IQR, 30–60). Lymphovascular and perineural invasion were identified in 131 (54.4%) and 99 (41.1%) patients, respectively. A median of 17 lymph nodes (IQR, 10–29) were examined, and 172 patients (71.4%) had at least 12 lymph nodes retrieved. R0 resection of the colorectal primary was achieved in 213 patients (88.4%).

An open approach was used in 188 patients (78.0%), emergency surgery was performed in 32 (13.3%), and a stoma was created during the index procedure in 120 (49.8%). Major postoperative complications, defined as Clavien–Dindo grade III or higher, occurred in 54 patients (22.4%). Eighteen patients (7.5%) underwent reoperation, and 10 patients (4.1%) died within 30 days.

Postoperative ICU admission was recorded in 200 patients (83.0%). Among ICU-admitted patients, the median ICU duration was 2 days (IQR, 1–3; range, 1–17). The median postoperative length of stay was 10 days (IQR, 7–15), and 62 patients (25.7%) remained hospitalized for more than 14 days.

### 3.2. Component Attainment and Textbook Outcome Reclassification

**Figure 2:**
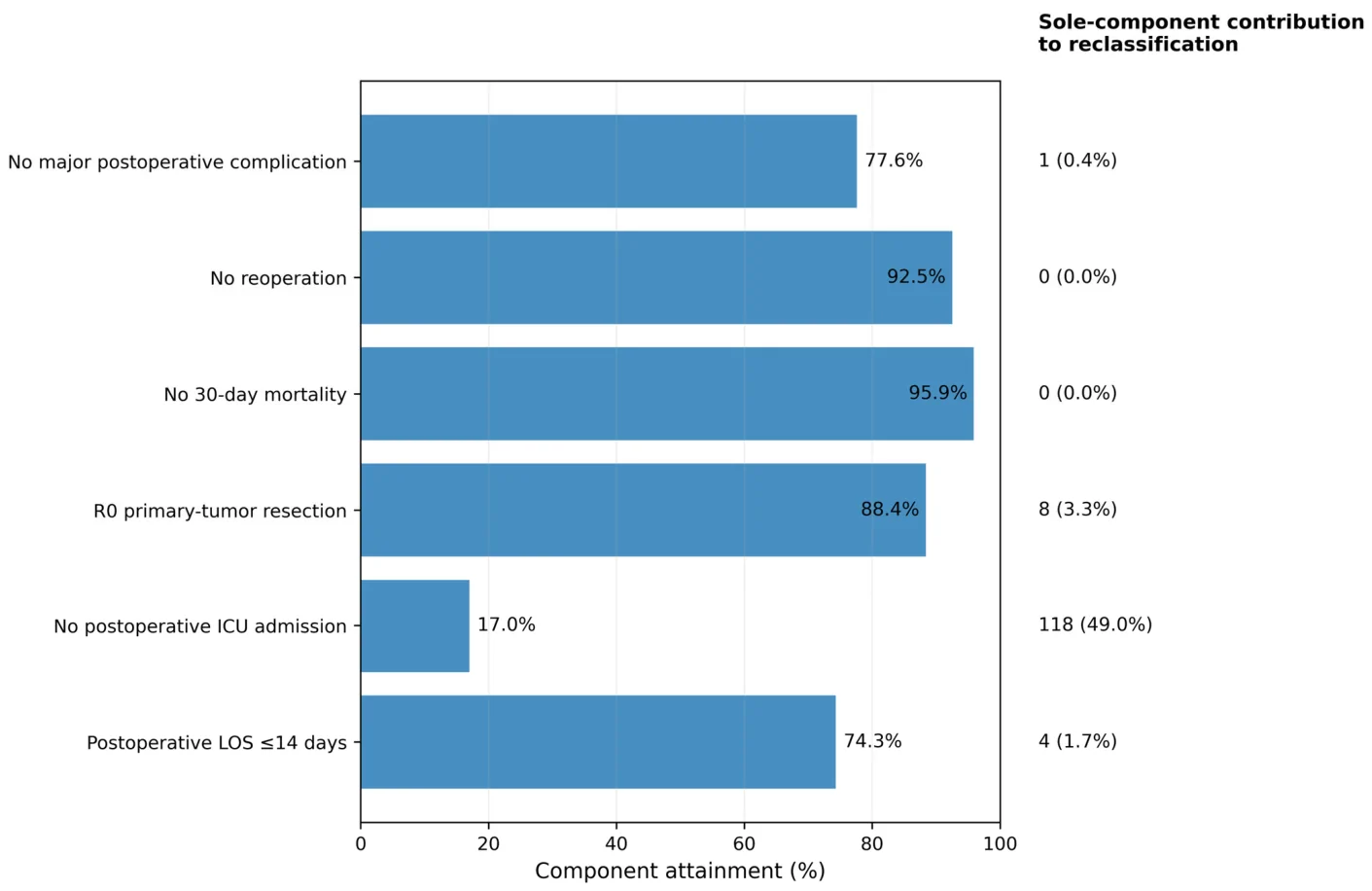
Component attainment and sole-component contribution to reclassification within the comprehensive textbook outcome construct. Horizontal bars show the proportion of patients fulfilling each component. The aligned text column shows the number and proportion of patients for whom the corresponding component alone prevented comprehensive TO achievement. ICU admission alone accounted for reclassification in 118 patients, compared with 8 for non-R0 primary-tumor resection, 4 for prolonged hospitalization, and 1 for major postoperative complication. These frequencies describe classification behavior under the observed component prevalence and should not be interpreted as direct measures of clinical importance. ICU, intensive care unit; LOS, length of stay; R0, microscopically margin-negative resection of the colorectal primary; TO, textbook outcome.

The attainment rates of the six comprehensive textbook outcome components are shown in Table 2 and Figure 2. Absence of a major complication was achieved in 187 patients (77.6%), absence of reoperation in 223 (92.5%), absence of 30-day mortality in 231 (95.9%), and R0 primary-tumor resection in 213 (88.4%). A postoperative length of stay of 14 days or less was achieved in 179 patients (74.3%). In contrast, only 41 patients (17.0%) avoided postoperative ICU admission.

Accordingly, the no-ICU criterion imposed a component-specific upper bound of 17.0% on comprehensive TO achievement, irrespective of the remaining components.

Core textbook outcome was achieved in 169 patients (70.1%; 95% confidence interval [CI], 64.1–75.6%), whereas comprehensive textbook outcome was achieved in 27 patients (11.2%; 95% CI, 7.8–15.8%). The paired absolute difference between the two definitions was 58.9 percentage points (95% CI, 52.6–64.9%; exact McNemar *p* < 0.001).

Patient-level reclassification across the nested definitions is illustrated in Figure 1. Twenty-seven patients (11.2%) achieved both definitions and were classified as having concordant TO achievement. A further 142 patients (58.9%) fulfilled all core TO criteria but failed comprehensive textbook outcome because of ICU admission and/or prolonged hospitalization. The remaining 72 patients (29.9%) had core TO failure. Clinical and pathological characteristics across the three patient-classification categories are presented in Supplementary Table S1.

Among the 142 patients with care-pathway-related reclassification:

- 118 (83.1%) failed exclusively because of postoperative ICU admission;
- 4 (2.8%) failed exclusively because of a length of stay exceeding 14 days;
- 20 (14.1%) failed because of both ICU admission and prolonged hospitalization.

The sole-component contribution analysis showed that ICU admission alone accounted for reclassification in 118 patients. The corresponding counts were eight for non-R0 primary-tumor resection, four for prolonged hospitalization, and one for major postoperative complication. Reoperation and 30-day mortality did not act alone because these events overlapped with at least one additional failure component. These frequencies describe classification behavior under the observed component prevalence and nested all-or-none structure and should not be interpreted as direct measures of clinical importance or intrinsic construct validity (Supplementary Table S2).

When the ICU component alone was omitted, the textbook outcome rate increased from 11.2% to 60.2%. In comparison, omission of the R0 component increased the rate to 14.5%, omission of the length-of-stay component to 12.9%, and omission of the major-complication component to 11.6% (Figure 2; Supplementary Table S2).

### 3.3. Concurrent Associations Across Postoperative ICU Duration

Postoperative ICU duration was distributed as follows: 41 patients had no ICU admission, 70 stayed for 1 day, 65 stayed for 2 days, and 65 required at least 3 ICU days. Clinical outcomes across these categories are summarized in Table 3.

The major complication rate increased from 4.9% among patients without ICU admission to 11.4% after a 1-day ICU stay, 16.9% after a 2-day stay, and 50.8% after an ICU duration of at least 3 days (global *p* < 0.001).

The corresponding core TO failure rates were:

- 24.4% after 0 ICU days;
- 18.6% after 1 ICU day;
- 20.0% after 2 ICU days;
- 55.4% after ≥3 ICU days (global *p* < 0.001).

Median postoperative length of stay increased from 8 days (IQR, 7–10) among patients without ICU admission and 8 days (IQR, 6–10) among those with a 1-day ICU stay to 12 days (IQR, 8–14) after 2 ICU days and 16 days (IQR, 12–23) after ≥3 ICU days (Kruskal–Wallis *p* < 0.001).

**Figure 3:**
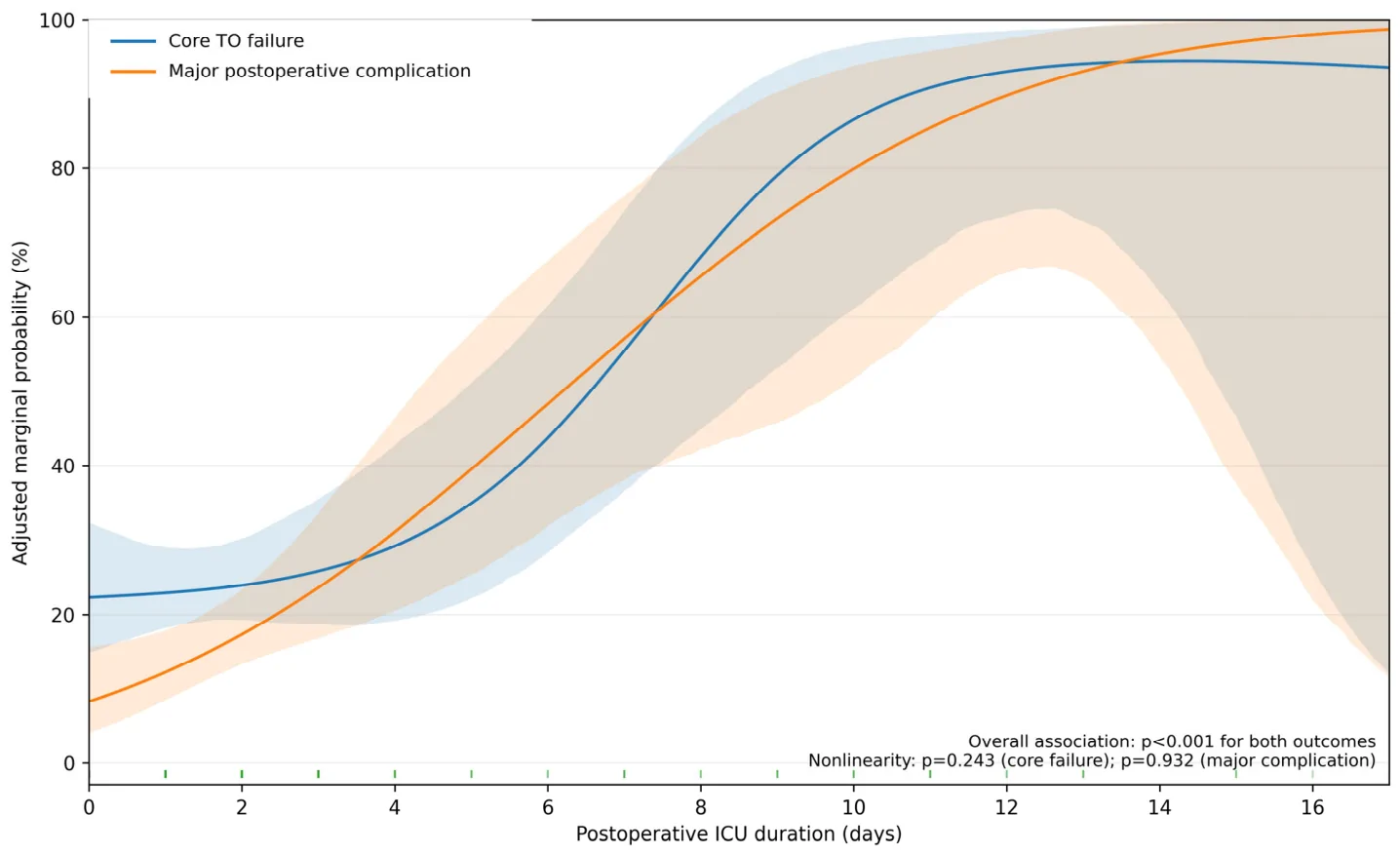
Adjusted association between continuous postoperative ICU duration and study outcomes. Curves represent marginal probabilities of core TO failure and major postoperative complications modeled using centered natural cubic splines with three degrees of freedom; shaded areas represent 95% confidence intervals. Rug marks indicate the observed ICU-duration distribution. Models were adjusted for age, ECOG performance status ≥2, synchronous metastatic disease, tumor perforation, and tumor-related obstruction. Overall associations were significant for both outcomes (both *p* < 0.001), with no evidence of departure from linearity for core TO failure (*p* = 0.243) or major postoperative complications (*p* = 0.932). Estimates at the longest ICU durations were based on relatively few observations and should be interpreted cautiously. ICU duration and postoperative complications arose during the same postoperative episode; the associations are concurrent rather than causal. ECOG, Eastern Cooperative Oncology Group; ICU, intensive care unit; TO, textbook outcome.

Prolonged hospitalization occurred in 9.8%, 2.9%, 24.6%, and 61.5% of the four ICU-duration groups, respectively (*p* < 0.001). Reoperation rates were 0%, 4.3%, 10.8%, and 12.3% (Fisher–Freeman–Halton exact *p* = 0.039), while 30-day mortality rates were 2.4%, 2.9%, 0%, and 10.8%, respectively (exact *p* = 0.017). When ICU duration was modeled continuously, each additional postoperative ICU day showed a concurrent association with higher odds of core TO failure (adjusted odds ratio (OR), 1.43; 95% CI, 1.25–1.63; *p* < 0.001) and major postoperative complications (adjusted OR, 1.63; 95% CI, 1.40–1.91; *p* < 0.001). Natural cubic spline analyses demonstrated significant overall associations for both outcomes (both *p* < 0.001), with no evidence of departure from linearity for core TO failure (*p* for nonlinearity = 0.243) or major postoperative complications (*p* for nonlinearity = 0.932). These findings support a graded relationship rather than a discrete duration change point (Figure 3 and Supplementary Table S8).

Compared with no ICU admission, the adjusted categorical estimates for 1-day (OR, 0.74; 95% CI, 0.25–2.16; *p* = 0.581) and 2-day ICU stays (OR, 0.59; 95% CI, 0.20–1.74; *p* = 0.339) did not reach statistical significance for core TO failure. However, the confidence intervals were wide and clinically relevant increases or decreases could not be excluded; these findings should not be interpreted as demonstrating equivalence with no ICU admission. In the exploratory categorical model, an ICU duration of ≥3 days was associated with core TO failure (OR, 4.38; 95% CI, 1.60–11.96; *p* = 0.004); however, this pooled category was not interpreted as a validated change point (Supplementary Table S12 and Supplementary Figure S1). The penalized sensitivity analyses further argued against a uniformly low-risk interpretation of short ICU stays. For nonfatal Clavien–Dindo grade III–IV complications, the 2-day category had a Firth OR of 5.40 (profile penalized-likelihood 95% CI, 1.13–53.02; *p* = 0.032), although the estimate was highly imprecise because of sparse events (Supplementary Table S9). The stability of the principal continuous-duration associations across prespecified sensitivity models is summarized in Table 4.

Case-mix-standardized probabilities showed the same pattern. The adjusted probabilities of core TO failure were 26.3%, 22.0%, 19.2%, and 53.3% for ICU durations of 0, 1, 2, and ≥3 days, respectively. The corresponding adjusted probabilities of major postoperative complication were 5.1%, 16.5%, 15.6%, and 49.3% (Supplementary Figure S3 and Supplementary Table S7).

The ≥3-day category showed the largest observed categorical difference but was created to combine sparse longer-duration observations and should not be interpreted as an empirically derived or biologically validated threshold. The exploratory ≥3-day versus 0–2-day analysis is reported in Supplementary Table S6 and was not used to identify or validate a duration cutoff.

### 3.4. Sensitivity of Textbook Outcome to the Operational Definition of ICU Utilization

**Figure 4:**
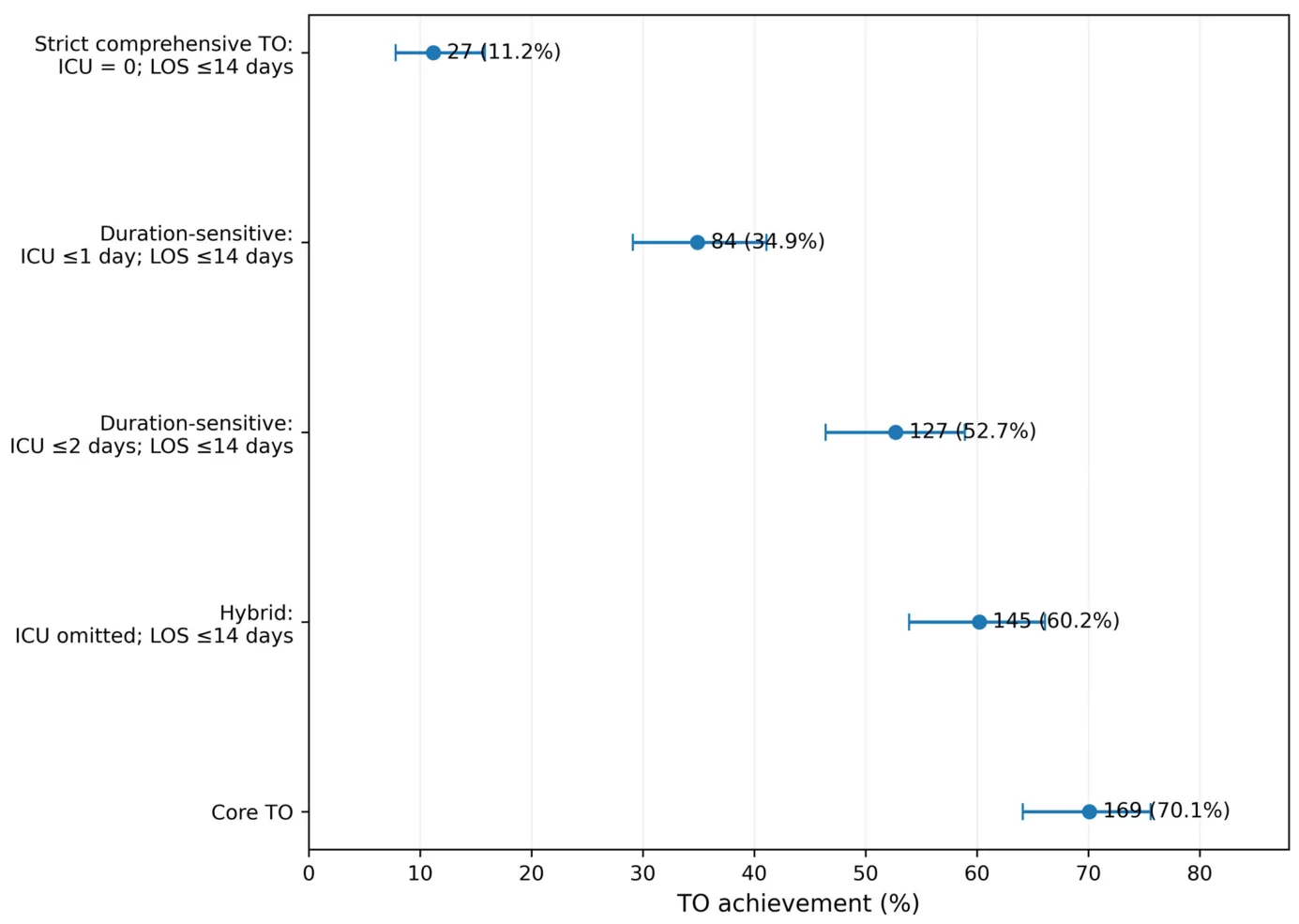
Sensitivity of textbook outcome achievement to alternative operational representations of postoperative ICU utilization. Points show TO achievement proportions and horizontal error bars show Wilson 95% confidence intervals. All scenarios are presented jointly as definition-sensitivity analyses; none represents a newly validated or universally recommended TO definition. TO, textbook outcome; ICU, intensive care unit; LOS, length of stay.

Textbook outcome achievement varied substantially according to the operational representation of postoperative ICU utilization (Figure 4 and Supplementary Table S3).

Under the strict comprehensive definition requiring no ICU admission and a length of stay ≤14 days, textbook outcome was achieved in 27 patients (11.2%). Allowing a single postoperative ICU day increased the achievement rate to 84 patients (34.9%). Allowing up to 2 ICU days increased the rate to 127 patients (52.7%).

When the ICU component was omitted but the 14-day length-of-stay criterion was retained, textbook outcome was achieved in 145 patients (60.2%). Core textbook outcome, omitting both ICU utilization and length of stay, was achieved in 169 patients (70.1%).

These scenarios were reported jointly as definition-sensitivity analyses; no alternative definition was selected according to the most favorable achievement rate.

The results were comparatively insensitive to reasonable changes in the prolonged-stay threshold when the strict no-ICU criterion was retained. Comprehensive textbook outcome rates were 9.1% using a 10-day threshold, 11.2% using the primary 14-day threshold, and 11.6% using the cohort-specific 75th percentile of 15 days (Supplementary Table S4). This contrasted with the substantially larger changes produced by altering the ICU component.

### 3.5. Internal Performance of Binary and Duration-Sensitive ICU Representations

Binary and duration-sensitive ICU representations were compared in otherwise identical case-mix-adjusted models (Table 5).

For core TO failure, the binary ICU model had an Akaike information criterion (AIC) of 260.70, an apparent area under the receiver-operating-characteristic curve (AUC) of 0.738, and a Brier score of 0.168. Replacing binary ICU status with the four-level duration variable reduced the AIC to 238.89, increased the apparent AUC to 0.816, and reduced the Brier score to 0.150. The duration-sensitive model provided significantly better fit than the binary model (likelihood-ratio χ^2^ = 25.82, 2 degrees of freedom; *p* < 0.001). After bootstrap correction for optimism, the corresponding AUCs were 0.714 for the binary model and 0.786 for the duration-sensitive model.

A similar pattern was observed for major postoperative complication. The binary model had an AIC of 213.96, an apparent AUC of 0.761, and a Brier score of 0.125. The duration-sensitive model had an AIC of 188.48, an apparent AUC of 0.859, and a Brier score of 0.107 (likelihood-ratio χ^2^ = 29.48; *p* < 0.001). The corresponding bootstrap-corrected AUCs were 0.741 for the binary model and 0.833 for the duration-sensitive model. Bootstrap convergence and optimism estimates are detailed in Supplementary Table S10.

These analyses evaluated concurrent postoperative associations and the information retained by alternative ICU representations. Because temporal ordering between complication onset and ICU duration could not be established, the coefficients should not be interpreted as causal effects, etiological estimates, or preoperative predictions. These improvements reflect internal performance within this dataset and do not constitute external validation or demonstrate transportability to centers with different ICU-admission policies. Because the indication for ICU care was unavailable, the duration-sensitive representation could not distinguish planned surveillance from unplanned rescue ICU use and should not be interpreted as a substitute for admission intent.

### 3.6. Sensitivity and Exploratory Analyses

The continuous ICU-duration estimates remained materially stable after expansion of the adjustment set (Supplementary Table S11). For core TO failure, the adjusted OR per additional ICU day was 1.43 (95% CI, 1.25–1.63) in the original model, 1.50 (95% CI, 1.30–1.73) after addition of ASA class ≥III and emergency operative status, 1.43 (95% CI, 1.25–1.63) after adjustment for calendar year, and 1.51 (95% CI, 1.31–1.75) after simultaneous addition of ASA class ≥III, emergency status, and calendar year. Restriction to patients with M0 disease yielded an OR of 1.42 (95% CI, 1.24–1.63). The corresponding estimates for major postoperative complications were 1.63 (95% CI, 1.40–1.91), 1.65 (95% CI, 1.40–1.94), 1.66 (95% CI, 1.40–1.99), 1.68 (95% CI, 1.40–2.02), and 1.62 (95% CI, 1.37–1.91), respectively; all *p* values were <0.001. Addition of surgical approach likewise did not materially change the continuous-duration estimates.

Emergency surgery was performed in 32 patients (13.3%). Postoperative ICU-admission rates varied across calendar years (global *p* = 0.042), but no monotonic linear trend was detected (OR per calendar-year increase, 0.92; 95% CI, 0.76–1.11; *p* = 0.386). Among ICU-admitted patients, ICU duration differed across calendar years (Kruskal–Wallis *p* < 0.001; Spearman ρ = 0.241; *p* < 0.001). The 2026 stratum represented the partial accrual period through 1 May 2026. Calendar-time analyses were exploratory and could not identify the specific institutional processes underlying annual variation.

Patient-level reclassification occurred in 61.2% of patients with M0 disease, 57.8% of patients with colon cancer, 60.4% of patients with rectal cancer, 59.0% of patients undergoing open surgery, and 58.5% of patients undergoing laparoscopic surgery (Supplementary Table S5 and Supplementary Figure S2). These subgroup analyses were descriptive and were not powered for formal interaction testing; similar percentages should not be interpreted as demonstrating statistical homogeneity or absence of effect modification.

## 4. Discussion

The principal finding of this study was that binary postoperative ICU admission exerted dominant influence on comprehensive TO classification in a single center where ICU utilization was frequent. Comprehensive and core TO rates differed by 58.9 percentage points, and ICU admission alone accounted for reclassification in 118 patients. This finding demonstrates poor compatibility between the no-ICU component and the local postoperative care pathway; it does not, by itself, establish that binary ICU admission is intrinsically invalid across other institutions. Actual ICU duration retained additional information about the postoperative course, with increasing duration showing a graded concurrent association with core TO failure and major postoperative complications. However, the categorical analyses did not establish equivalence between 0-, 1-, and 2-day stays, and the ≥3-day grouping should be regarded as an exploratory presentation rather than a validated clinical threshold. Because ICU utilization and complications evolved during the same postoperative episode, all adjusted estimates represent concurrent associations rather than causal effects.

Published TO achievement rates provide important context but cannot be interpreted independently of the definitions that generated them. A recent meta-analysis reported a pooled colorectal TO rate of approximately 55%, while a prospective multicenter cohort and the LASRE trial reported achievement rates of approximately 52% and 75%, respectively [2,3,6]. However, these studies differed in population, operative setting, complication threshold, oncological criteria, readmission window, length-of-stay threshold, and inclusion of ICU utilization. Systematic reviews have subsequently confirmed that TO remains a family of related composite constructs rather than a single standardized endpoint [6,7,8]. Our results extend this literature by demonstrating that definitional heterogeneity does not merely change aggregate rates between publications; it can change the classification of most patients within the same cohort. The comprehensive and core rates should therefore not be interpreted as competing estimates of the “true” quality of care. They measure different constructs: the comprehensive construct combines the core criteria with care-pathway components, whereas core TO is a minimal analytical comparator based on major postoperative complications, reoperation, mortality, and primary-specimen margin status. Neither construct should be interpreted as the uniquely correct measure of surgical quality.

This distinction exposes both the appeal and the vulnerability of the all-or-none architecture. TO sets a high standard because every component must be achieved, but equal veto power does not imply equal prevalence, clinical importance, patient-valued importance, or susceptibility to institutional policy [1,4,5,18,19,20]. Because only 17.0% of patients avoided postoperative ICU admission, the no-ICU criterion imposed a mathematical ceiling of 17.0% on comprehensive TO before the remaining components were considered. The high prevalence of ICU utilization therefore necessarily gave this component substantial numerical influence. This finding demonstrates classification dominance in the present cohort; it does not independently establish the clinical unimportance of other components or the intrinsic invalidity of ICU admission as a TO criterion. Component prevalence, sole-component contribution, clinical importance, and construct validity are related but distinct properties.

Major postoperative complications, reoperation, and mortality rarely acted as sole-component contributors because these severe outcomes frequently overlapped. Their low sole-component contribution therefore reflects structural dependence within the composite rather than limited clinical importance. Core TO also retains the unweighted all-or-none architecture: major complication, reoperation, mortality, and non-R0 primary-tumor resection remain binary components with equal classification power. Removing ICU admission and LOS isolated the influence of two care-pathway components but did not resolve the broader equal-weighting limitation of composite endpoints. Core TO should therefore be interpreted as an analytical comparator rather than a proposed replacement score.

Postoperative ICU admission is particularly sensitive to this problem because it is not a homogeneous event. ICU care may be planned before surgery for enhanced observation of an older or high-risk patient, instituted immediately after a complex procedure because ward-level monitoring is considered insufficient, or required unexpectedly because of physiological deterioration. Planned and unplanned ICU admission after colorectal cancer surgery have different clinical determinants [10]. Routine overnight ICU observation has even been associated with fewer adverse postoperative outcomes in selected older colorectal cancer patients [21], whereas unplanned ICU admission following colon cancer surgery identifies a markedly high-risk postoperative course [13]. Studies of postoperative cancer populations have likewise shown substantial heterogeneity in the timing, indication, and outcome of ICU admission [22,23]. A binary component necessarily collapses these distinct scenarios into a single failure state. It therefore risks classifying precautionary surveillance and rescue treatment as clinically equivalent events.

The high rate of ICU utilization in our center should not be used either to defend or to condemn the local care pathway. Rather, it created an informative setting in which to test the transportability of the binary ICU component. If a metric intended for interinstitutional benchmarking becomes almost unattainable in a hospital where postoperative critical-care monitoring is frequently used, the relevant question is not whether the hospital should receive a more favorable score. The relevant question is whether the component preserves the same clinical meaning across different organizational settings. A transportable quality component should remain interpretable when monitoring policies, ward capabilities, nurse-to-patient ratios, ICU availability, and discharge practices vary. In the present cohort, the binary ICU component showed limited compatibility with the local postoperative care pathway because it combined 1 day and as many as 17 days of ICU utilization within the same failure category. This observation evaluates local classification behavior and should not be extrapolated to centers with substantially lower ICU-admission rates.

The continuous analyses provided the clearest evidence regarding ICU duration. Each additional ICU day showed a concurrent association with higher odds of core TO failure and major postoperative complications, and spline analyses supported a graded relationship without evidence of a discrete nonlinear change point. The categorical estimates for 1- and 2-day stays were imprecise and did not establish equivalence with no ICU admission. This uncertainty was reinforced by the penalized nonfatal-complication analysis, in which the 2-day category had a Firth OR of 5.40 but a very wide profile penalized-likelihood 95% CI of 1.13–53.02. Accordingly, short ICU stays cannot be characterized uniformly as low-risk observation on the basis of these data.

The ≥3-day category showed the largest observed categorical difference, but it pooled sparse longer-duration observations and should not be interpreted as a biological or clinical change point. Duration-sensitive representations showed better internal fit, discrimination, and probabilistic performance than binary admission, but these differences do not constitute external validation. ICU duration may be a consequence, correlate, or marker of an evolving complication rather than its cause; the appropriate interpretation is therefore one of concurrent clinical information rather than causal effect. A recent colorectal cohort similarly associated prolonged ICU stay with sepsis, prolonged mechanical ventilation, venous thrombosis, and other markers of postoperative severity [24], but that study does not validate a universal duration threshold.

The definition-sensitivity analysis further illustrates why our results should not be reduced to a recommendation to “allow” a specified number of ICU days. Comprehensive TO increased from 11.2% under the strict no-ICU definition to 34.9% when 1 ICU day was permitted and to 52.7% when up to 2 days were permitted. Omitting the ICU component while retaining the 14-day LOS criterion yielded a rate of 60.2%, whereas omission of both care-pathway components yielded the core TO rate of 70.1%. These values demonstrate measurement instability, not a hierarchy of progressively better definitions. Selecting the scenario that produces the most favorable rate would simply replace one arbitrary decision rule with another. Similarly, interpreting the observed change at 3 days as a definitive universal cutoff would be premature and vulnerable to optimism. ICU duration should preferentially be retained as a continuous or ordinal measure, with clinically interpretable categories used for description and externally validated sensitivity analyses. The practical contribution of this study is therefore not the proposal of a new universal TO definition, but evidence that the operational representation of ICU care requires explicit justification.

The contrast between ICU admission and LOS was also informative. Changing the prolonged-stay threshold from 10 to 14 or 15 days altered comprehensive TO by only a few percentage points, whereas changing the ICU representation produced differences exceeding 40 percentage points. Thus, although both variables are potentially center-sensitive, ICU admission was the principal driver of classification in this cohort. LOS nevertheless remains an imperfect surrogate for recovery. Even after uncomplicated colorectal surgery, discharge may be delayed by stoma education, rehabilitation requirements, caregiver availability, social support, patient confidence, or access to step-down facilities [25]. For this reason, LOS should not be dismissed as irrelevant, but its meaning should be separated from major postoperative complications and reported with a clearly defined starting point, threshold, and healthcare context.

These observations do not invalidate TO as a concept. Risk-adjusted hospital TO rates have been associated with long-term outcomes across high-risk oncological procedures, supporting the value of TO as a summary measure of the cancer-care episode [26]. The present findings instead define conditions under which that value may be compromised. TO is more defensible for benchmarking when its components are clinically coherent, consistently available, sufficiently specific, and transportable across institutions. Previous colorectal work has shown that identifying “best-performing” hospitals depends materially on the selected quality indicators and analytical approach [27].

Our data suggest that future colorectal TO reports should disclose the exact operational definition and time window of every component, component-specific attainment rates, sole-component contributions, local ICU-admission practices, and sensitivity analyses using duration or planned/unplanned status where available. Readmission windows, whether stoma-related components distinguish planned or protective stomas from unplanned stomas, lymph-node-yield requirements, and procedure-specific margin definitions should likewise be reported explicitly because these components vary substantially across existing TO constructs. This is not a replacement TO score. It is a transparency framework that allows readers to determine whether a reported TO rate is driven by hard clinical failure or by the organization of postoperative care.

The strengths of this study include use of a fully verified real-world cohort, complete ascertainment of the primary composite components, and patient-level measurement of cumulative postoperative ICU days rather than reliance on a binary administrative code. The nested design allowed an exact paired comparison within the same patients, thereby eliminating between-cohort case-mix differences from the primary reclassification analysis. In addition to conventional component-attainment reporting, we quantified sole-component contributions, constructed a definition-sensitivity ladder, and compared binary and duration-sensitive ICU representations in otherwise identical case-mix models. Reclassification percentages were directionally similar across exploratory metastatic-status, tumor-location, and operative-approach subgroups, although no formal interaction testing was performed. Finally, optimism correction reduced model performance estimates, as expected, but did not remove the advantage of the duration-sensitive representation.

Several limitations require emphasis. First, this was a retrospective single-center study conducted in a setting where 83.0% of patients received postoperative ICU care. This practice pattern made the measurement question observable but limits external validity; the magnitude of reclassification cannot be extrapolated to institutions with substantially lower ICU utilization or different bed-management policies. Second, planned or prophylactic ICU monitoring could not be distinguished consistently from unplanned ICU admission or transfer prompted by postoperative deterioration. These pathways differ in indication, temporal sequence, severity, and prognosis, and their combination introduces clinically important exposure misclassification into both the binary and duration-sensitive analyses. Although duration retained more information than a yes/no variable, it could not reconstruct why ICU care was initiated and should be considered a partial refinement rather than a substitute for admission intent or a measure of ICU appropriateness. Third, the timing of complication onset relative to ICU admission, transfer, and discharge could not be established consistently. ICU duration and adverse outcomes therefore represent temporally interdependent features of the same postoperative episode, precluding causal or etiological interpretation.

Fourth, the continuous ICU-duration estimates remained materially similar after adjustment for ASA class, emergency operative status, calendar year, and surgical approach and after restriction to M0 disease; nevertheless, residual confounding remains possible. Detailed operative duration, blood loss, transfusion requirement, multivisceral resection, organ-support intensity, provider-level decision making, ward-monitoring capacity, weekend staffing, and patient-level enhanced recovery after surgery (ERAS) adherence were not available with sufficient completeness for analysis. Calendar year was evaluated, but this variable could not identify specific changes in ICU policy, bed availability, staffing, ERAS implementation, or coronavirus disease 2019 (COVID-19)-era care pathways. Fifth, the cohort included heterogeneous colon and rectal procedures, synchronous metastatic disease, and protocol-eligible palliative resections. The M0 sensitivity analysis was directionally consistent but did not fully remove heterogeneity related to operative intent, and R0 referred only to the colorectal primary rather than complete metastatic clearance.

Sixth, core TO was a minimal analytical comparator rather than a complete oncological quality measure. It did not include lymph-node yield, procedure-specific rectal margin quality, readmission, or stoma-related outcomes and retained the general limitations of an unweighted all-or-none composite. Seventh, sparse outcome cells produced wide confidence intervals in the categorical and Firth models. Penalization reduced small-sample bias but did not eliminate substantial uncertainty regarding the magnitude of several estimates. Eighth, multiple secondary, sensitivity, subgroup, and alternative-threshold analyses were performed without formal multiplicity correction. These analyses were exploratory, and isolated *p* values should be interpreted cautiously. Ninth, subgroup analyses were descriptive and were not powered for interaction testing; similar percentages do not demonstrate statistical homogeneity. Finally, spline estimates at the longest ICU durations were based on few observations and wide confidence intervals and should not be interpreted with the same precision as estimates near the center of the observed distribution.

## 5. Conclusions

In this single center with frequent postoperative ICU utilization, binary ICU admission dominated comprehensive TO classification and discarded information contained in actual ICU duration. Increasing ICU duration showed a graded concurrent association with adverse postoperative outcomes, but the present data do not establish equivalence between short ICU stays and no ICU admission or identify a universal duration threshold. The findings demonstrate local classification dominance and context sensitivity rather than the intrinsic invalidity of the ICU component.

Before TO is used for interinstitutional benchmarking, studies should report exact component definitions, component prevalence, sole-component contributions, and local care-pathway characteristics. Multicenter external validation across institutions with different ICU policies, including planned versus unplanned admission status and standardized perioperative-pathway data, is necessary.

## Figures and Tables

**Table 1:** Baseline Demographic, Clinical, Operative, Pathological, and Postoperative Characteristics of the Study Cohort.

Variable	Overall Cohort (*n* = 241)
**Demographic and clinical characteristics**	
Age, years	66 (55–72)
Male sex	165 (68.5%)
ASA class ≥ III	45 (18.7%)
ECOG performance status ≥ 2	83 (34.4%)
Diabetes mellitus	74 (30.7%)
Hypertension	98 (40.7%)
Coronary artery disease	51 (21.2%)
Chronic obstructive pulmonary disease	16 (6.6%)
**Tumor and pathological characteristics**	
Tumor-related obstruction	68 (28.2%)
Tumor perforation	41 (17.0%)
Rectal tumor location	106 (44.0%)
Clinical T3–4 category	198 (82.2%)
Final pathological nodal involvement (pN/ypN positive)	108 (44.8%)
Synchronous metastatic disease	27 (11.2%)
Pathology-reported maximum dimension, mm	43 (30–60)
Lymphovascular invasion	131 (54.4%)
Perineural invasion	99 (41.1%)
Harvested lymph nodes, *n*	17 (10–29)
At least 12 lymph nodes examined	172 (71.4%)
R0 primary tumor resection	213 (88.4%)
**Operative characteristics**	
Operative urgency	
— Elective	209 (86.7%)
— Emergency	32 (13.3%)
Surgical approach	
— Open	188 (78.0%)
— Laparoscopic	53 (22.0%)
Stoma formation	120 (49.8%)
**Postoperative outcomes**	
Major postoperative complication (Clavien–Dindo ≥ III)	54 (22.4%)
Reoperation within 30 days	18 (7.5%)
Postoperative ICU admission	200 (83.0%)
ICU duration among admitted patients, days	2 (1–3)
Postoperative length of stay, days	10 (7–15)
Length of stay > 14 days	62 (25.7%)
30-day mortality	10 (4.1%)

Data are presented as median (interquartile range) or *n* (%). R0 refers to microscopically margin-negative resection of the colorectal primary specimen, irrespective of synchronous distant metastatic disease. In patients with ypT0 pathological complete response, a non-zero pathology-reported dimension represents the tumor bed or fibrotic scar rather than viable residual tumor. Abbreviations: ASA, American Society of Anesthesiologists; ECOG, Eastern Cooperative Oncology Group; ICU, intensive care unit; IQR, interquartile range; pN, pathological nodal category following upfront surgery; ypN, post-treatment pathological nodal category following neoadjuvant treatment.

**Table 2:** Component Attainment, Textbook Outcome Achievement, and Patient-Level Reclassification.

Measure	*n*	%	95% CI/Statistical Result
No major postoperative complication	187	77.6	71.9–82.4
No reoperation	223	92.5	88.5–95.2
No 30-day mortality	231	95.9	92.5–97.7
R0 primary-tumor resection	213	88.4	83.7–91.8
No postoperative ICU admission	41	17.0	12.8–22.3
Postoperative length of stay ≤ 14 days	179	74.3	68.4–79.4
Core TO achieved	169	70.1	64.1–75.6
Comprehensive TO achieved	27	11.2	7.8–15.8
Patient-level reclassification attributable to care-pathway components	142	58.9	52.6–64.9; exact McNemar *p* < 0.001
Concordant TO achievement	27	11.2	—
Care-pathway-related reclassification	142	58.9	—
— ICU-only reclassification	118	49.0	—
— LOS-only reclassification	4	1.7	—
— Combined ICU-plus-LOS reclassification	20	8.3	—
Core TO failure	72	29.9	—

Wilson confidence intervals are reported for proportions. Because the constructs were nested, the paired difference equals the reclassification proportion; comprehensive and core TO achievement were compared using the exact McNemar test. Percentages for ICU-only, LOS-only, and combined ICU-plus-LOS reclassification use the entire cohort as the denominator. Within the 142 reclassified patients, these mechanisms accounted for 83.1%, 2.8%, and 14.1%, respectively. Reoperation and grade V mortality are structurally nested within the Clavien–Dindo classification; sole-component contribution frequencies describe composite-classification behavior and should not be interpreted as measures of clinical importance. Abbreviations: CI, confidence interval; ICU, intensive care unit; LOS, length of stay; TO, textbook outcome.

**Table 3:** Clinical Outcomes According to Postoperative ICU Duration.

ICU Duration	*n*	LOS, Median (IQR), Days	Major Postoperative Complication, *n* (%)	Reoperation, *n* (%)	30-Day Mortality, *n* (%)	Core TO Failure, *n* (%)	LOS > 14 Days, *n* (%)
0 days	41	8 (7–10)	2 (4.9%)	0 (0.0%)	1 (2.4%)	10 (24.4%)	4 (9.8%)
1 day	70	8 (6–10)	8 (11.4%)	3 (4.3%)	2 (2.9%)	13 (18.6%)	2 (2.9%)
2 days	65	12 (8–14)	11 (16.9%)	7 (10.8%)	0 (0.0%)	13 (20.0%)	16 (24.6%)
≥3 days	65	16 (12–23)	33 (50.8%)	8 (12.3%)	7 (10.8%)	36 (55.4%)	40 (61.5%)
Global *p* value	—	<0.001	<0.001	0.039	0.017	<0.001	<0.001

Postoperative LOS was compared using the Kruskal–Wallis test. Major postoperative complications, core TO failure, and LOS > 14 days were compared using the Pearson chi-square test. Reoperation and 30-day mortality were compared using the Fisher–Freeman–Halton exact test. The categorical ICU-duration groups were used for descriptive presentation. The ≥3-day category pooled sparse longer-duration observations and should not be interpreted as a validated threshold. Abbreviations: ICU, intensive care unit; IQR, interquartile range; LOS, length of stay; TO, textbook outcome.

**Table 4:** Stability of the Concurrent Association Between Postoperative ICU Duration and Study Outcomes.

Outcome	Model	Adjusted OR per Additional ICU Day	95% CI	*p* Value
Core TO failure	Original adjustment	1.43	1.25–1.63	<0.001
Core TO failure	+ASA ≥ III, emergency status, and calendar year	1.51	1.31–1.75	<0.001
Core TO failure	M0 disease only	1.42	1.24–1.63	<0.001
Major postoperative complication	Original adjustment	1.63	1.40–1.91	<0.001
Major postoperative complication	+ASA ≥ III, emergency status, and calendar year	1.68	1.40–2.02	<0.001
Major postoperative complication	M0 disease only	1.62	1.37–1.91	<0.001

The original adjustment set included age per 10-year increase, ECOG performance status ≥ 2, synchronous metastatic disease, tumor perforation, and tumor-related obstruction. Expanded models additionally included ASA class ≥ III, emergency operative status, and calendar year. Calendar year was modeled continuously. M0-restricted models used the original adjustment set without metastatic status because that variable was constant. Associations represent concurrent postoperative associations and should not be interpreted as causal effects or preoperative predictions. Detailed sensitivity analyses are presented in Supplementary Table S11. Abbreviations: ASA, American Society of Anesthesiologists; CI, confidence interval; ECOG, Eastern Cooperative Oncology Group; ICU, intensive care unit; M0, absence of synchronous distant metastatic disease; OR, odds ratio; TO, textbook outcome.

**Table 5:** Comparison of Internal Model Performance Under Binary and Duration-Sensitive ICU Representations.

Outcome	ICU Representation	AIC	Apparent AUC	Optimism-Corrected AUC	Brier Score	Nested-Model Comparison
Core TO failure	Binary: any ICU admission	260.70	0.738	0.714	0.168	—
Core TO failure	Duration-sensitive: 0, 1, 2, and ≥3 days	238.89	0.816	0.786	0.150	LR χ^2^ = 25.82; df = 2; *p* < 0.001
Major postoperative complication	Binary: any ICU admission	213.96	0.761	0.741	0.125	—
Major postoperative complication	Duration-sensitive: 0, 1, 2, and ≥3 days	188.48	0.859	0.833	0.107	LR χ^2^ = 29.48; df = 2; *p* < 0.001

Binary and duration-sensitive representations were evaluated in otherwise identical case-mix-adjusted logistic regression models. Lower AIC and Brier scores indicate better model fit and probabilistic accuracy, respectively. Optimism correction used 500 bootstrap attempts. Successful fits were 500/500 for both core TO failure models, 483/500 for the binary major-complication model, and 488/500 for the duration-sensitive major-complication model. These estimates describe internal performance within the present dataset and do not constitute external validation or establish transportability to institutions with different ICU-admission policies. Abbreviations: df, degrees of freedom; AIC, Akaike information criterion; AUC, area under the receiver-operating-characteristic curve; ICU, intensive care unit; LR, likelihood ratio; TO, textbook outcome.

## Data Availability

The data supporting the findings of this study are available from the corresponding author upon reasonable request, subject to institutional approval and applicable data-protection regulations. The data are not publicly available because of institutional data governance and patient confidentiality requirements.

## Supplementary Materials

The following supporting information can be downloaded at https://www.mdpi.com/article/10.3390/jcm15176809/s1. Figure S1. Adjusted associations with core TO failure. Points represent adjusted odds ratios and horizontal bars represent 95% confidence intervals from the duration-sensitive multivariable logistic regression model. ICU duration was represented by indicator variables for 1 day, 2 days, and ≥3 days, with 0 ICU days as the reference category. Models were adjusted for age, ECOG performance status ≥2, synchronous metastatic disease, tumor perforation, and tumor-related obstruction. The associations are concurrent and should not be interpreted as causal effects. ECOG, Eastern Cooperative Oncology Group; ICU, intensive care unit; OR, odds ratio; TO, textbook outcome. Figure S2. Patient-level reclassification across exploratory clinical subgroups. Reclassification was defined as achievement of core TO with failure of comprehensive TO because of postoperative ICU admission and/or prolonged hospitalization. Points represent subgroup-specific reclassification proportions, and horizontal bars represent Wilson 95% confidence intervals. These analyses were exploratory and were not powered for formal interaction testing. ICU, intensive care unit; TO, textbook outcome. Figure S3. Adjusted marginal probabilities according to categorical postoperative ICU duration. Points represent marginal probabilities standardized over the observed distributions of age, ECOG performance status, synchronous metastatic disease, tumor perforation, and tumor-related obstruction; error bars represent 95% confidence intervals obtained by parametric simulation. The 0-, 1-, 2-, and ≥3-day categories were used for descriptive presentation. The ≥3-day category pooled sparse longer-duration observations and should not be interpreted as a validated threshold. ECOG, Eastern Cooperative Oncology Group; ICU, intensive care unit; TO, textbook outcome. Figure S4. Conceptual directed acyclic graph for the ICU-duration analyses. The directed acyclic graph summarizes the assumed relationships among baseline patient factors, acute tumor-related presentation, operative characteristics, center- and provider-level care factors, evolving postoperative physiological severity, ICU admission and duration, and postoperative outcomes. Evolving postoperative severity was conceptualized as a common determinant of ICU duration and adverse postoperative outcomes. No causal pathway from ICU duration to postoperative complications was assumed. ICU, intensive care unit. Table S1. Clinical and pathological characteristics across patient-classification categories. Table S2. Component failure frequency, sole-component contribution, and leave-one-component-out analysis. Table S3. Sensitivity of TO achievement to alternative ICU representations. Table S4. Sensitivity analysis using alternative prolonged-stay thresholds. Table S5. Patient-level reclassification across exploratory clinical subgroups. Table S6. Exploratory ICU-duration threshold analysis. Table S7. Adjusted marginal outcome probabilities according to postoperative ICU duration. Table S8. Continuous and spline ICU-duration analyses. Table S9. Bias-reduced and nonfatal-complication sensitivity analyses. Table S10. Bootstrap convergence and optimism-corrected discrimination estimates. Table S11. Sensitivity analyses of the continuous ICU-duration association and calendar-time variation. Table S12. Maximum-likelihood categorical ICU-duration models for core TO failure and major postoperative complications.
