# Supplementary material for: Beyond Binary ICU Admission: Duration-Sensitive Evaluation and Patient-Level Reclassification of Textbook Outcome After Colorectal Cancer Surgery
Source: J Clin Med. 2026 Sep 2;15(17):6809. doi: 10.3390/jcm15176809 (PMC13566375; doi:10.3390/jcm15176809)
Supplement: Supplementary file 1 [file jcm-15-06809-s001.zip › supplementary table s1.pdf]

Supplementary Table S1. Clinical and Pathological Characteristics Across Patient-Classification Categories

Variable	Concordant TO Achievement (n=27)	Care-Pathway-Related Reclassification (n=142)	Core TO Failure (n=72)	Global p Value
Age, years	59 (46–68)	64 (53–71)	67 (62–76)	0.003
Pathology-reported maximum dimension, mm	42 (32–50)	40 (25–60)	50 (40–73)	0.002
Harvested lymph nodes, n	15 (11–18)	19 (11–29)	17 (9–30)	0.072
Positive lymph nodes, n	0 (0–1)	0 (0–2)	1 (0–3)	0.005
Male sex	19 (70.4%)	103 (72.5%)	43 (59.7%)	0.168
ECOG performance status ≥2	4 (14.8%)	39 (27.5%)	40 (55.6%)	<0.001
ASA class ≥III	3 (11.1%)	25 (17.6%)	17 (23.6%)	0.361
Tumor-related obstruction	12 (44.4%)	26 (18.3%)	30 (41.7%)	<0.001
Tumor perforation	8 (29.6%)	7 (4.9%)	26 (36.1%)	<0.001
Rectal tumor location	9 (33.3%)	64 (45.1%)	33 (45.8%)	0.535
Synchronous metastatic disease	0 (0.0%)	11 (7.7%)	16 (22.2%)	0.001
Open surgical approach	17 (63.0%)	111 (78.2%)	60 (83.3%)	0.094
Stoma formation	5 (18.5%)	67 (47.2%)	48 (66.7%)	<0.001
Final pathological nodal involvement	8 (29.6%)	58 (40.8%)	42 (58.3%)	0.013
Lymphovascular invasion	12 (44.4%)	70 (49.3%)	49 (68.1%)	0.018
Perineural invasion	13 (48.1%)	52 (36.6%)	34 (47.2%)	0.236

Data are presented as median (interquartile range) or n (%). Continuous variables were compared using the Kruskal–Wallis test. Categorical variables were compared using the Fisher–Freeman–Halton exact test or Pearson chi-square test, as appropriate.

These classification-category comparisons were exploratory and supportive. No multiplicity correction was applied, and the small concordant-achievement group limits precision.

Abbreviations: ASA, American Society of Anesthesiologists; ECOG, Eastern Cooperative Oncology Group; TO, textbook outcome.
