# Supplementary material for: Beyond Binary ICU Admission: Duration-Sensitive Evaluation and Patient-Level Reclassification of Textbook Outcome After Colorectal Cancer Surgery
Source: J Clin Med. 2026 Sep 2;15(17):6809. doi: 10.3390/jcm15176809 (PMC13566375; doi:10.3390/jcm15176809)
Supplement: Supplementary file 1 [file jcm-15-06809-s001.zip › supplementary table s10.pdf]

Supplementary Table S10. Bootstrap Convergence and Optimism-Corrected Discrimination Estimates

**Outcome**	**ICU Representation**	**Apparent AUC**	**Mean Optimism**	**Optimism-Corrected AUC**	**Successful Fits / Attempts**
Core TO failure	Binary: any ICU admission	0.738	0.024	0.714	500 / 500
Core TO failure	Duration-sensitive: 0, 1, 2, and ≥3 days	0.816	0.030	0.786	500 / 500
Major postoperative complication	Binary: any ICU admission	0.761	0.020	0.741	483 / 500
Major postoperative complication	Duration-sensitive: 0, 1, 2, and ≥3 days	0.859	0.026	0.833	488 / 500

For each successfully fitted bootstrap sample, optimism was calculated as the difference between the AUC within the bootstrap sample and the AUC obtained when the bootstrap-fitted model was applied to the original cohort; mean optimism was subtracted from the apparent AUC. Abbreviations: AUC, area under the receiver-operating-characteristic curve; ICU, intensive care unit; TO, textbook outcome.
