# Supplementary material for: Beyond Binary ICU Admission: Duration-Sensitive Evaluation and Patient-Level Reclassification of Textbook Outcome After Colorectal Cancer Surgery
Source: J Clin Med. 2026 Sep 2;15(17):6809. doi: 10.3390/jcm15176809 (PMC13566375; doi:10.3390/jcm15176809)
Supplement: Supplementary file 1 [file jcm-15-06809-s001.zip › supplementary table s11.pdf]

Supplementary Table S11. Sensitivity Analyses of the Continuous ICU-Duration Association and Calendar-Time Variation

Panel A. Stability of the Adjusted Association per Additional Postoperative ICU Day

Outcome	Sensitivity Model	Adjusted OR per Additional ICU Day	95% CI	p Value	Interpretation
Core TO failure	Original adjustment	1.43	1.25–1.63	<0.001	Reference model
Core TO failure	+ ASA class ≥III	1.45	1.27–1.65	<0.001	Estimate materially unchanged
Core TO failure	+ Emergency operative status	1.48	1.28–1.70	<0.001	Estimate materially unchanged
Core TO failure	+ ASA class ≥III and emergency status	1.50	1.30–1.73	<0.001	Estimate materially unchanged
Core TO failure	+ Calendar year (continuous)	1.43	1.25–1.63	<0.001	Estimate materially unchanged
Core TO failure	+ ASA class ≥III, emergency status, and calendar year	1.51	1.31–1.75	<0.001	Estimate materially unchanged
Core TO failure	+ Surgical approach	1.43	1.25–1.63	<0.001	Estimate materially unchanged
Core TO failure	M0 disease only	1.42	1.24–1.63	<0.001	Direction and magnitude preserved
Major postoperative complication	Original adjustment	1.63	1.40–1.91	<0.001	Reference model
Major postoperative complication	+ ASA class ≥III	1.63	1.39–1.91	<0.001	Estimate materially unchanged
Major postoperative complication	+ Emergency operative status	1.65	1.40–1.94	<0.001	Estimate materially unchanged
Major postoperative complication	+ ASA class ≥III and emergency status	1.65	1.40–1.94	<0.001	Estimate materially unchanged
Major postoperative complication	+ Calendar year (continuous)	1.66	1.40–1.99	<0.001	Estimate materially unchanged
Major postoperative complication	+ ASA class ≥III, emergency status, and calendar year	1.68	1.40–2.02	<0.001	Estimate materially unchanged
Major postoperative complication	+ Surgical approach	1.63	1.40–1.91	<0.001	Estimate materially unchanged
Major postoperative complication	M0 disease only	1.62	1.37–1.91	<0.001	Direction and magnitude preserved

Panel B. Distribution of Postoperative ICU Utilization and Outcomes by Calendar Year

Calendar Year	Patients, n	Postoperative ICU Admission, n (%)	ICU Duration Among Admitted Patients, Median (IQR), Days	Major Postoperative Complication, n (%)	Core TO Failure, n (%)
2020	20	17 (85.0%)	2 (1–3)	6 (30.0%)	7 (35.0%)
2021	30	23 (76.7%)	1 (1–2.5)	9 (30.0%)	13 (43.3%)
2022	40	34 (85.0%)	1 (1–1.8)	9 (22.5%)	10 (25.0%)
2023	41	39 (95.1%)	2 (1–2.5)	7 (17.1%)	7 (17.1%)
2024	43	38 (88.4%)	2 (2–3)	3 (7.0%)	6 (14.0%)
2025	48	33 (68.8%)	2 (2–3)	16 (33.3%)	22 (45.8%)
2026	19	16 (84.2%)	4 (2–5.3)	4 (21.1%)	7 (36.8%)

The original adjustment set included age per 10-year increase, ECOG performance status ≥2, synchronous metastatic disease, tumor perforation, and tumor-related obstruction.

Calendar year was modeled continuously. Emergency operative status was coded as emergency versus elective; 32 operations (13.3%) were emergency.

Annual ICU-admission rates varied across years (Pearson χ^2 p=0.042), but no monotonic linear trend was detected (OR per calendar-year increase, 0.92; 95% CI, 0.76–1.11; p=0.386).

Among ICU-admitted patients, ICU duration differed across calendar years (Kruskal–Wallis p<0.001; Spearman ρ=0.241; p<0.001). The 2026 stratum represents the partial accrual period through 1 May 2026.

These analyses were exploratory. No multiplicity correction was applied; interpretation focused on stability of the ICU-duration estimate rather than isolated p values.

Abbreviations: ASA, American Society of Anesthesiologists; CI, confidence interval; ECOG, Eastern Cooperative Oncology Group; ICU, intensive care unit; IQR, interquartile range; M0, absence of synchronous distant metastatic disease; OR, odds ratio; TO, textbook outcome.
