# Supplementary material for: Beyond Binary ICU Admission: Duration-Sensitive Evaluation and Patient-Level Reclassification of Textbook Outcome After Colorectal Cancer Surgery
Source: J Clin Med. 2026 Sep 2;15(17):6809. doi: 10.3390/jcm15176809 (PMC13566375; doi:10.3390/jcm15176809)
Supplement: Supplementary file 1 [file jcm-15-06809-s001.zip › supplementary table s12.pdf]

Supplementary Table S12. Maximum-Likelihood Categorical ICU-Duration Models for Core TO Failure and Major Postoperative Complications

Outcome	Variable	Adjusted OR	95% CI	p Value
Core TO failure	ICU duration: 1 day vs. 0 days	0.74	0.25–2.16	0.581
Core TO failure	ICU duration: 2 days vs. 0 days	0.59	0.20–1.74	0.339
Core TO failure	ICU duration: ≥3 days vs. 0 days	4.38	1.60–11.96	0.004
Core TO failure	Age, per 10-year increase	1.28	0.94–1.74	0.119
Core TO failure	ECOG performance status ≥2	2.76	1.25–6.08	0.012
Core TO failure	Synchronous metastatic disease	5.06	1.75–14.69	0.003
Core TO failure	Tumor perforation	5.99	1.93–18.58	0.002
Core TO failure	Tumor-related obstruction	0.67	0.25–1.82	0.436
Major postoperative complication	ICU duration: 1 day vs. 0 days	5.28	0.86–32.49	0.073
Major postoperative complication	ICU duration: 2 days vs. 0 days	4.82	0.83–27.99	0.079
Major postoperative complication	ICU duration: ≥3 days vs. 0 days	45.30	7.71–266.28	<0.001
Major postoperative complication	Age, per 10-year increase	1.20	0.85–1.71	0.308
Major postoperative complication	ECOG performance status ≥2	2.83	1.13–7.14	0.027
Major postoperative complication	Synchronous metastatic disease	1.03	0.30–3.49	0.962
Major postoperative complication	Tumor perforation	6.66	1.93–22.97	0.003
Major postoperative complication	Tumor-related obstruction	1.71	0.60–4.86	0.314

Separate multivariable logistic regression models were adjusted for age per 10-year increase, ECOG performance status ≥2, synchronous metastatic disease, tumor perforation, and tumor-related obstruction.

These maximum-likelihood estimates were affected by sparse outcome cells, particularly for the major-complication model. Bias-reduced Firth estimates are reported in Supplementary Table S9. Confidence-interval width, rather than statistical significance alone, should guide interpretation. Associations are concurrent and should not be interpreted as causal effects or preoperative predictions.

Abbreviations: CI, confidence interval; ECOG, Eastern Cooperative Oncology Group; ICU, intensive care unit; OR, odds ratio; TO, textbook outcome.
