# Supplementary material for: Beyond Binary ICU Admission: Duration-Sensitive Evaluation and Patient-Level Reclassification of Textbook Outcome After Colorectal Cancer Surgery
Source: J Clin Med. 2026 Sep 2;15(17):6809. doi: 10.3390/jcm15176809 (PMC13566375; doi:10.3390/jcm15176809)
Supplement: Supplementary file 1 [file jcm-15-06809-s001.zip › supplementary table s2.pdf]

Supplementary Table S2. Component Failure Frequency, Sole-Component Contribution, and Leave-One-Component-Out Analysis

Component	Component Failure Frequency	Sole-Component Contribution to Reclassification	Comprehensive TO Rate if Component Omitted
No major postoperative complication	54 (22.4%)	1 (0.4%)	28 (11.6%)
No reoperation	18 (7.5%)	0 (0.0%)	27 (11.2%)
No 30-day mortality	10 (4.1%)	0 (0.0%)	27 (11.2%)
R0 primary-tumor resection	28 (11.6%)	8 (3.3%)	35 (14.5%)
No postoperative ICU admission	200 (83.0%)	118 (49.0%)	145 (60.2%)
Postoperative length of stay ≤14 days	62 (25.7%)	4 (1.7%)	31 (12.9%)

A sole-component contribution was defined as failure of one component in a patient who fulfilled every other comprehensive TO criterion.
The leave-one-component-out rate represents the number and proportion of patients who would have achieved comprehensive TO had that component not been included.
Sole-component contribution describes classification behavior under the observed component prevalence and should not be interpreted as a direct measure of clinical importance or construct validity.
Abbreviations: ICU, intensive care unit; TO, textbook outcome.
