# Supplementary material for: Beyond Binary ICU Admission: Duration-Sensitive Evaluation and Patient-Level Reclassification of Textbook Outcome After Colorectal Cancer Surgery
Source: J Clin Med. 2026 Sep 2;15(17):6809. doi: 10.3390/jcm15176809 (PMC13566375; doi:10.3390/jcm15176809)
Supplement: Supplementary file 1 [file jcm-15-06809-s001.zip › supplementary table s3.pdf]

Supplementary Table S3. Sensitivity of Textbook Outcome Achievement to Alternative ICU Representations

Scenario	TO Achieved, n (%)	95% CI
Strict comprehensive TO: ICU = 0 days and LOS ≤14 days	27 (11.2%)	7.8–15.8
Duration-sensitive scenario: ICU ≤1 day and LOS ≤14 days	84 (34.9%)	29.1–41.1
Duration-sensitive scenario: ICU ≤2 days and LOS ≤14 days	127 (52.7%)	46.4–58.9
Hybrid scenario: ICU component omitted; LOS ≤14 days retained	145 (60.2%)	53.9–66.1
Core TO	169 (70.1%)	64.1–75.6

All scenarios are presented jointly as definition-sensitivity analyses. None represents a newly validated or universally preferred TO definition.

Abbreviations: CI, confidence interval; ICU, intensive care unit; LOS, length of stay; TO, textbook outcome.
