# Supplementary material for: Beyond Binary ICU Admission: Duration-Sensitive Evaluation and Patient-Level Reclassification of Textbook Outcome After Colorectal Cancer Surgery
Source: J Clin Med. 2026 Sep 2;15(17):6809. doi: 10.3390/jcm15176809 (PMC13566375; doi:10.3390/jcm15176809)
Supplement: Supplementary file 1 [file jcm-15-06809-s001.zip › supplementary table s4.pdf]

Supplementary Table S4. Sensitivity Analysis Using Alternative Prolonged-Stay Thresholds

Length-of-Stay Threshold	Comprehensive TO Achieved, n (%)
LOS ≤10 days	22 (9.1%)
LOS ≤14 days	27 (11.2%)
LOS ≤15 days	28 (11.6%)

The strict no-ICU criterion was retained in all scenarios. The 14-day threshold was the primary analysis threshold; 10 and 15 days represented the cohort median and cohort-specific 75th percentile, respectively.
Abbreviations: ICU, intensive care unit; LOS, length of stay; TO, textbook outcome.
