# Supplementary material for: Beyond Binary ICU Admission: Duration-Sensitive Evaluation and Patient-Level Reclassification of Textbook Outcome After Colorectal Cancer Surgery
Source: J Clin Med. 2026 Sep 2;15(17):6809. doi: 10.3390/jcm15176809 (PMC13566375; doi:10.3390/jcm15176809)
Supplement: Supplementary file 1 [file jcm-15-06809-s001.zip › supplementary table s5.pdf]

Supplementary Table S5. Patient-Level Reclassification Across Exploratory Clinical Subgroups

Exploratory Subgroup	n	Core TO, n (%)	Comprehensive TO, n (%)	Reclassified, n (%)	Reclassification 95% CI
Overall cohort	241	169 (70.1%)	27 (11.2%)	142 (58.9%)	52.6–64.9
M0 disease	214	158 (73.8%)	27 (12.6%)	131 (61.2%)	54.5–67.5
Colon cancer	135	96 (71.1%)	18 (13.3%)	78 (57.8%)	49.3–65.8
Rectal cancer	106	73 (68.9%)	9 (8.5%)	64 (60.4%)	50.9–69.2
Open surgery	188	128 (68.1%)	17 (9.0%)	111 (59.0%)	51.9–65.8
Laparoscopic surgery	53	41 (77.4%)	10 (18.9%)	31 (58.5%)	45.1–70.7

Reclassification was defined as achievement of core TO with failure of comprehensive TO because of postoperative ICU admission and/or prolonged hospitalization.

These analyses were exploratory and descriptive and were not powered for formal interaction testing. Similar percentages do not demonstrate statistical homogeneity or absence of effect modification.

Abbreviations: CI, confidence interval; ICU, intensive care unit; M0, absence of synchronous distant metastatic disease; TO, textbook outcome.
