# Supplementary material for: Beyond Binary ICU Admission: Duration-Sensitive Evaluation and Patient-Level Reclassification of Textbook Outcome After Colorectal Cancer Surgery
Source: J Clin Med. 2026 Sep 2;15(17):6809. doi: 10.3390/jcm15176809 (PMC13566375; doi:10.3390/jcm15176809)
Supplement: Supplementary file 1 [file jcm-15-06809-s001.zip › supplementary table s6.pdf]

Supplementary Table S6. Exploratory ICU-Duration Threshold Analysis

Outcome	Adjusted OR for ICU Duration ≥3 vs. 0–2 Days	95% CI	p Value
Core TO failure	5.94	2.90–12.18	<0.001
Major postoperative complication	11.35	4.92–26.17	<0.001

Models were adjusted for age, ECOG performance status ≥2, synchronous metastatic disease, tumor perforation, and tumor-related obstruction.

The ≥3-day threshold analysis was exploratory and should not be interpreted as establishing a universal ICU-duration cutoff.

Abbreviations: CI, confidence interval; ECOG, Eastern Cooperative Oncology Group; ICU, intensive care unit; OR, odds ratio; TO, textbook outcome.
