# Supplementary material for: Beyond Binary ICU Admission: Duration-Sensitive Evaluation and Patient-Level Reclassification of Textbook Outcome After Colorectal Cancer Surgery
Source: J Clin Med. 2026 Sep 2;15(17):6809. doi: 10.3390/jcm15176809 (PMC13566375; doi:10.3390/jcm15176809)
Supplement: Supplementary file 1 [file jcm-15-06809-s001.zip › supplementary table s7.pdf]

Supplementary Table S7. Adjusted Marginal Outcome Probabilities According to Postoperative ICU Duration

ICU Duration	Adjusted Core TO Failure Probability, %	95% CI	Adjusted Major Postoperative Complication Probability, %	95% CI
0 days	26.3	16.2–40.4	5.1	1.4–16.0
1 day	22.0	14.5–32.2	16.5	9.8–26.4
2 days	19.2	12.5–29.5	15.6	9.8–24.9
≥3 days	53.3	42.6–64.2	49.3	38.4–60.3

Marginal probabilities were standardized over the observed distributions of age, ECOG performance status, synchronous metastatic disease, tumor perforation, and tumor-related obstruction.

Confidence intervals were obtained through parametric simulation from the fitted duration-sensitive logistic regression models.

The categorical groups were used for descriptive presentation and do not establish a validated duration threshold.

Abbreviations: CI, confidence interval; ECOG, Eastern Cooperative Oncology Group; ICU, intensive care unit; TO, textbook outcome.
