# Supplementary material for: Beyond Binary ICU Admission: Duration-Sensitive Evaluation and Patient-Level Reclassification of Textbook Outcome After Colorectal Cancer Surgery
Source: J Clin Med. 2026 Sep 2;15(17):6809. doi: 10.3390/jcm15176809 (PMC13566375; doi:10.3390/jcm15176809)
Supplement: Supplementary file 1 [file jcm-15-06809-s001.zip › supplementary table s8.pdf]

Supplementary Table S8. Continuous and Spline ICU-Duration Analyses

Outcome	Adjusted OR per Additional ICU Day	95% CI	p Value	Spline Overall LR χ^2 (df=3)	Overall p Value	Nonlinearity LR χ^2 (df=2); p
Core TO failure	1.43	1.25–1.63	<0.001	46.50	<0.001	2.83; 0.243
Major postoperative complication	1.63	1.40–1.91	<0.001	68.52	<0.001	0.14; 0.932
Nonfatal major postoperative complication (Clavien–Dindo III–IV)	1.51	1.32–1.72	<0.001	58.74	<0.001	1.31; 0.518

Models were adjusted for age per 10-year increase, ECOG performance status ≥2, synchronous metastatic disease, tumor perforation, and tumor-related obstruction.

Spline analyses used centered natural cubic splines with three degrees of freedom. No statistically detectable departure from linearity was identified.

The estimates represent concurrent postoperative associations rather than causal effects.

Abbreviations: CI, confidence interval; ICU, intensive care unit; LR, likelihood ratio; OR, odds ratio; TO, textbook outcome.
