# Supplementary material for: Beyond Binary ICU Admission: Duration-Sensitive Evaluation and Patient-Level Reclassification of Textbook Outcome After Colorectal Cancer Surgery
Source: J Clin Med. 2026 Sep 2;15(17):6809. doi: 10.3390/jcm15176809 (PMC13566375; doi:10.3390/jcm15176809)
Supplement: Supplementary file 1 [file jcm-15-06809-s001.zip › supplementary table s9.pdf]

Supplementary Table S9. Bias-Reduced and Nonfatal-Complication Sensitivity Analyses

Outcome	ICU Duration Category	Firth OR	Profile Penalized-Likelihood 95% CI	Penalized LR p Value
Core TO failure	1 day vs. 0 days	0.74	0.26–2.13	0.572
Core TO failure	2 days vs. 0 days	0.60	0.21–1.73	0.343
Core TO failure	≥3 days vs. 0 days	4.01	1.56–11.08	0.004
Major postoperative complication	1 day vs. 0 days	4.20	0.90–27.26	0.070
Major postoperative complication	2 days vs. 0 days	3.86	0.88–23.99	0.076
Major postoperative complication	≥3 days vs. 0 days	31.36	7.40–200.84	<0.001
Nonfatal major postoperative complication (Clavien–Dindo III–IV)	1 day vs. 0 days	3.46	0.65–35.49	0.156
Nonfatal major postoperative complication (Clavien–Dindo III–IV)	2 days vs. 0 days	5.40	1.13–53.02	0.032
Nonfatal major postoperative complication (Clavien–Dindo III–IV)	≥3 days vs. 0 days	19.11	4.34–182.77	<0.001

Firth penalized logistic regression was used to reduce small-sample and separation bias in the categorical ICU-duration models.

Penalization reduced bias but did not remove substantial uncertainty; the wide profile penalized-likelihood confidence intervals should receive greater interpretive weight than whether p crossed 0.05.

Models used the same adjustment set as the corresponding maximum-likelihood models.

Abbreviations: CI, confidence interval; ICU, intensive care unit; LR, likelihood ratio; OR, odds ratio; TO, textbook outcome.
